# Structural connectivity profile supports laterality of the salience network

**DOI:** 10.1002/hbm.24769

**Published:** 2019-08-21

**Authors:** Yaodan Zhang, Xinjun Suo, Hao Ding, Meng Liang, Chunshui Yu, Wen Qin

**Affiliations:** ^1^ From the Department of Radiology and Tianjin Key Laboratory of Functional Imaging Tianjin Medical University General Hospital Tianjin China; ^2^ School of Medical Imaging Tianjin Medical University Tianjin China

**Keywords:** aging, diffusion magnetic resonance imaging, dorsal anterior cingulate cortex, frontal insula cortex, laterality, salience network, structural connectivity

## Abstract

The salience network (SN) is mainly involved in detecting and filtering multimodal salient stimuli, and mediating the switch between the default mode network and central executive network. Early studies have indicated a right‐sided dominance in the functional organization of the SN; however, the anatomical basis of the functional lateralization remains unclear. Here, we hypothesized that the structural connectivity profile between the frontoinsular cortex (FIC) and dorsal anterior cingulate cortex (dACC), which are two core hubs of the SN, is also dominant in the right hemisphere. Based on diffusion and resting‐state functional magnetic resonance imaging (rfMRI) of adult healthy volunteers in independent datasets, we found a stable right‐sided laterality of both the FIC‐dACC structural and functional connectivity in both the human connectome project cohort and a local Chinese cohort. Furthermore, a significant effect of aging on the integrity of the right FIC‐dACC structural connectivity was also identified. The right‐sided laterality of the structural organization of the SN may help us to better understand the functional roles of the SN in the normal human brain.

## 1 INTRODUCTION

The salience network (SN) is mainly involved in detecting and filtering multimodal salient stimuli, and mediating the switch between the default mode network (DMN) and central executive network (CEN; Fox, Snyder, Vincent, & Raichle, 2007; Sridharan, Levitin, & Menon, 2008), in which the frontoinsular cortex (FIC) and dorsal anterior cingulate cortex (dACC) are two core hubs (Goulden et al., 2014; Seeley et al., 2007; Zhou et al., 2010). In early studies, the organization and roles of the SN were mainly discovered from a functional perspective (He et al., 2013; Uddin et al., 2013). For example, task‐based functional magnetic resonance imaging (fMRI) studies have consistently reported that the FIC and dACC are among the most frequently coactivated regions by salient stimuli from diverse sensory modalities (Levy & Wagner, 2011; Sridharan et al., 2008; Uddin, 2015). Additionally, these two SN hubs are dynamically coupled at both task and resting‐state conditions (Chen, Chen, Liu, & Shi, 2016; Kim et al., 2016; Kucyi, Hodaie, & Davis, 2012; Sridharan et al., 2008). In contrast to the functional interactions, reports about the structural relationships between the two areas are fewer. Several studies had identified that the von Economo neurons (VENs) specifically exist in the FIC and dACC of great apes and humans (Allman et al., 2010; Cobos & Seeley, 2015); furthermore, early evidence indicates that these two regions are interconnected with each other by VENs projections (Allman et al., 2010; Craig, 2009). Based on diffusion magnetic resonance imaging (dMRI), a recent study successfully tracked the FIC‐dACC fibers trajectory in vivo, and found that the integrity of FIC‐dACC structural connectivity can predict the activity of the DMN in traumatic brain during a stop‐signal task (Bonnelle et al., 2012). These findings suggested that the structural and functional connectivities between the FIC and dACC play important roles in the functionality of the SN.

Early studies have reported a right‐sided dominance of the SN in both functional organization and roles (Seeley et al., 2007). For example, the right rather than the left FIC is predominantly activated by salient stimuli (Craig, 2002; Sridharan et al., 2008). Additionally, the right FIC rather than the left had higher net causal flow to the dACC, CEN, and DMN during auditory event segmentation, visual oddball attention tasks, and task‐free resting‐state (Sridharan et al., 2008). Dynamic causal modeling analyses also demonstrated that salient input into the SN was most likely via the right FIC during the Simon task (Ham, Leff, de Boissezon, Joffe, & Sharp, 2013). Furthermore, the intrinsic functional couplings of the SN are also stronger and broader in the right FIC and dACC than those in the left side, as identified by functional connectivity (FC; Cauda et al., 2011) and independent component analysis (ICA; Seeley et al., 2007). Significant associations were also identified between the individual's fluid reasoning capacity and the gray matter volume (GMV) and regional homogeneity (ReHo) in only the right FIC (Yuan et al., 2012). However, it remains unclear whether anatomical organization underlies the functional laterality of the SN.

Early studies have demonstrated that the functional coupling between brain areas is closely associated with direct or indirect structural connectivity (Honey et al., 2009; Teipel et al., 2010). Moreover, several human and monkey histological studies reported right‐sided dominance of the amount of VENs in the FIC and dACC (Allman et al., 2010; Evrard, Forro, & Logothetis, 2012; Gonzalez‐Acosta, Escobar, Casanova, Pimienta, & Buritica, 2018). Thus, we hypothesized that the right laterality in both the regional activation and functional coupling of the SN may have a structural basis. Specifically, the structural connectivity profile between the FIC and dACC, two core hubs of SN, may be also dominant in the right hemisphere. This hypothesis was supported by one recent study showing that the structural connectivity profile of the arcuate fasciculus can characterize the left laterality of the language functional network (Sreedharan, Menon, James, Kesavadas, & Thomas, 2015). Finally, early studies had reported that normal aging is closely associated with reduced multiple dimensional cognitive functions that relate to the salient network, such as alerting (Jennings, Dagenbach, Engle, & Funke, 2007), task‐switching (Verhaeghen & Cerella, 2002), and conflict processing (West, 2004), and so on. The FC within the right SN, and between the right FIC and the CEN/DMN had also been reported to be negatively correlated with aging (He et al., 2013; He et al., 2014). Thus, we also hypothesized that normal aging can decrease the integrity of the structural connectivity of the right FIC‐dACC.

To validate these hypotheses, we constructed the FIC‐dACC structural connectivity and calculated the structural laterality based on dMRI, which is a noninvasive and popular technique to trace the large‐scale fiber bundles in the brain (Basser, Pajevic, Pierpaoli, Duda, & Aldroubi, 2000; Wakana, Jiang, Nagae‐Poetscher, van Zijl, & Mori, 2004). Given that the traditional second‐order diffusion tensor model cannot resolve the complex crossing fibers, in the present study, we adopted an advanced reconstruction technique named generalized q‐sampling imaging (GQI), which estimates the spin distribution function (SDF) directly from diffusion MR signals using either shell or grid sampling schemes, and can reliably reconstruct multiple fiber orientations within one single voxel (Yeh & Tseng, 2010; Yeh, Verstynen, Wang, Fernandez‐Miranda, & Tseng, 2013). It has been shown in the ISMRM 2015 Tractography challenge that the GQI‐based method has achieved the highest valid connections (92%) among 96 fiber‐tracking methods (Maier‐Hein et al., 2017). The laterality of FC between the two hubs was also calculated using the resting‐state fMRI (rfMRI) data. Based on the structural and FC profiles from independent datasets from the human connectome project (HCP) and two Chinese cohorts, we tried to determine: (a) whether the functional laterality of the SN can be replicated in different cohorts; (b) whether the structural connectivity profile of the SN is also reliably lateralized in the right hemisphere; and (c) whether normal aging decreases the integrity of the structural connectivity of the right FIC‐dACC.

## 2 MATERIALS AND METHODS

### 2.1 Subjects

#### 2.1.1 Discovery cohorts

This study included 100 normal, unrelated adults (age: 22–35 years old, including 50 males) from the WU‐Minn HCP Consortium. The detailed subject recruitment information for the HCP cohort is provided at the website (https://www.humanconnectome.org/study/hcp-young-adult/document/1200-subjects-data-release). The HCP project acquires high‐quality multimodal MR imaging data using advanced custom‐made MR equipment and imaging protocols (Van Essen et al., 2013). For example, the dMRI data were collected with high spatial resolution (1.25 mm^3^) and high‐angular resolution (288 diffusion directions, and multiple diffusion weights which *b* values =1,000, 2,000, 3,000 s/m^2^), and rfMRI data were also collected with high spatial (2 mm^3^) and temporal resolutions (720 ms per frame). In combination with state‐of‐the‐art reconstruction algorithms, these high‐quality data can dramatically improve the accuracy of fiber reconstruction and FC calculation between the FIC and dACC (Sotiropoulos et al., 2013; Ugurbil et al., 2013). As a result, the analysis and interpretation procedure of the HCP dataset was considered as the cardinal line in the present study.

#### 2.1.2 Validation cohorts

We first acquired the MR data of a cohort of 50 healthy, unrelated Chinese young individuals (age: 18–25 years old, including 25 males) from a local institute to validate the laterality of FIC‐dACC as revealed by HCP cohorts. The inclusion criteria were as follows: (a) 18–30 years old; (b) right handedness evaluated using the Edinburgh Handedness Inventory (Oldfield, 1971); (c) Han nationality; (d) no history of alcohol abuse using the Alcohol Use Disorders Identification Test (score > 7; Saunders, Aasland, Babor, de la Fuente, & Grant, 1993) or drug abuse; (e) no history or suspicion of psychiatric disorder based on the Mini‐International Neuropsychiatric Interview (Sheehan et al., 1998); (f) no severe neurological and somatic disorders; (g) no MRI contraindications; 8) no smoking habit (lifetime smoking <20 cigarettes); (h) no color discrimination disorder; (i) unpregnancy; (j) no history of family psychiatric disorders.

Additionally, datasets of a cohort of 112 healthy Chinese elder individuals (age: 40–75 years old, including 61 males and 51 females) were also gathered to test the effect of normal aging on the integrity of right FIC‐dACC structural connectivity. It should be noted that these datasets came from the healthy control groups of two early stroke studies by our group (Jiang et al., 2017; Liu, Qin, Zhang, Zhang, & Yu, 2015). The exclusion criteria were as follows: (a) left‐handedness; (b) any visible brain abnormalities on MR images; (c) histories of any other psychiatric or neurologic disorders; (d) MRI contraindications; (e) MRI artifacts in the data. The demographic information for all of the groups was provided in Table 1.

**Table 1:** Demographic information of the recruited subjects

Cohorts	Subsets	No. of subjects	Age (years)	Gender
HCP cohort	‐	100	20–25	50 males
Chinese young cohort	‐	50	18–25	25 males
Chinese elder cohort	Subset 1	27	41–67	13 males
	Subset 2	35	40–75	21 males
	Subset 3	28	40–72	15 males
	Subset 4	22	47–74	12 males

**Figure 1:**
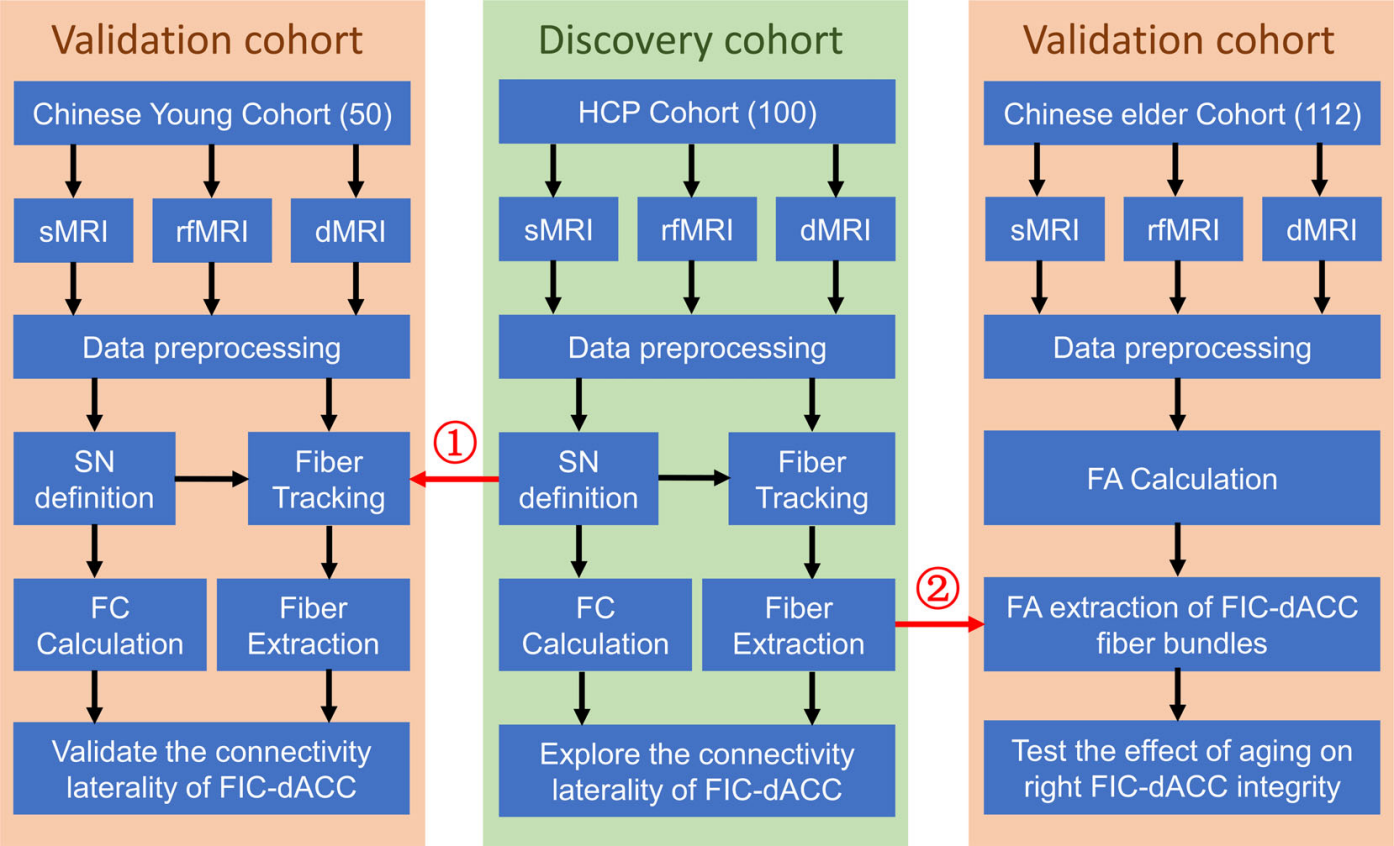
Flow diagram of the study. ① The FIC and dACC defined by HCP rfMRI dataset were used as the seeds to track the fiber bundles in the Chinese young cohort. ② The fiber probability map created using the HCP dMRI dataset was used to extract the FA values along the right FIC‐dACC fiber trajectory in the Chinese elder cohort. Abbreviations: dACC, dorsal anterior cingulate cortex; dMRI, diffusion magnetic resonance imaging; FA, fractional anisotropy; FIC, frontoinsular cortex; HCP, human connectome project; FIC, frontoinsular cortex; rfMRI, resting‐state functional magnetic resonance imaging; SN, salience network

The datasets of the two Chinese cohorts were obtained under the permission by the Ethics Committee of Tianjin Medical University General Hospital, and written informed consent was obtained from each participant. For better understanding the purpose of each cohort and the analyses pipelines, we provided a schematic diagram in Figure 1.

### 2.2 Imaging acquisition

#### 2.2.1 HCP dataset

The HCP dataset was downloaded from the HCP website (https://www.humanconnectome.org/study/hcp-young-adult), which was obtained using a custom‐made 3.0 T WU‐Minn‐Ox HCP scanner with very strong magnetic field gradients (100 mT/m) along with optimized pulse sequences (Glasser et al., 2016). In the present study, dMRI, rfMRI, and high‐resolution 3D T1‐weighted structural MRI (sMRI) data were used: The rfMRI data were used to construct the SN, evaluate the laterality of FC, and validate its reliability across runs; the dMRI was used to evaluate the laterality of structural connectivity; the sMRI data was for spatial normalization. The website (http://protocols.humanconnectome.org/HCP/3T/imaging-protocols.html) provided imaging parameters in detail.

#### 2.2.2 Chinese datasets

MRI data for the Chinese cohorts were obtained using four different MRI scanners, including one GE Signa HDx scanner (GE Healthcare, Milwaukee, WI), two GE Discovery MR750 scanners, and a Simens Magnetom Trio Tim MR scanner (Siemens, Erlangen, Germany). Three image modalities were used in this study, dMRI, rfMRI, and sMRI. The key imaging parameters of all scanners are shown in Table 2.

**Table 2:** Imaging parameters for the enrolled datasets

Dataset/scanner	Modality	Sequence	TR/TE/TI (ms)	Flip angle (degree)	FOV (mm)	Matrix	Thickness/gap (mm)	Slices	Other parameters
HCP/WU‐Minn‐Ox HCP scanner	sMRI	MPRAGE	2400/2.14/1000	8	224 × 224	320 × 320	0.7/0	260	‐
	dMRI	SE‐EPI	5520/89.5	78	210 × 180	168 × 144	1.25/0	111	Multiband factor = 3 b = 1,000, 2,000, 3,000 s/mm^2^ Num. b0 = 6 Num. directions = 288
	rfMRI	GRE‐EPI	720/33.1	52	208 × 180	104 × 90	2/0	72	Time points = 1,200
Chinese young cohort/MR750	sMRI	BRAVO	8.14/3.17/450	12	256 × 256	256 × 256	1/0	188	‐
	dMRI	SE‐EPI	7100/60.5	90	256 × 256	128 × 128	2/0	70	b = 1,000 s/mm^2^ Num. b0 = 5 Num. directions = 64
	rfMRI	GRE‐EPI	2000/30	90	220 × 220	64 × 64	3/1	36	Time points = 180
Chinese elder subset 1/Trio Tim	sMRI	MPRAGE	2000/2.26/900	9	256 × 232	256 × 232	1/0	192	‐
	dMRI	SE‐EPI	6000/85	90	256 × 256	128 × 128	3/0	45	b = 1,000 s/mm^2^ Num. b0 = 1 Num. directions = 64
Chinese elder subset 2/MR750	sMRI	BRAVO	8.14/3.17/450	12	256 × 256	256 × 256	1/0	188	‐
	dMRI	SE‐EPI	7100/60.5	90	256 × 256	128 × 128	2/0	70	b = 1,000 s/mm^2^ Num. b0 = 10 Num. Directions = 64
Chinese elder subset 3/MR750	sMRI	BRAVO	8.16/3.18/450	12	256 × 256	256 × 256	1/0	188	‐
	dMRI	SE‐EPI	7100/60.5	90	256 × 256	128 × 128	2/0	70	b = 1,000 s/mm^2^ Num. b0 = 10 Num. directions = 64
Chinese elder subset 4/Signa HDx	sMRI	BRAVO	8.16/3.12/450	13	256 × 256	256 × 256	1/0	176	‐
	dMRI	SE‐EPI	10,000/64.7	90	256 × 256	128 × 128	3/0	45	b = 1,000 s/mm^2^ Num. b0 = 3 Num. Directions = 55

### 2.3 Data preprocessing

#### 2.3.1 HCP dataset

The downloaded HCP rfMRI dataset had been preprocessed using the minimal procedures, including gradient distortion correction, motion correction, spatial normalization to the Montreal Neurological Institute (MNI) space, high‐pass filtering, and denoising based on the ICA‐fix algorithm (Glasser et al., 2013; Van Essen et al., 2013). The detailed procedures are provided in the website (https://www.humanconnectome.org/storage/app/media/documentation/s1200/HCP_S1200_Release_Reference_Manual.pdf). The minimal preprocessed rfMRI data were subjected to nuisance regression including the following regressors: rigid motion parameters and their time derivatives, spike frames with framewise displacement (Power, Barnes, Snyder, Schlaggar, & Petersen, 2012) higher than 0.5, the signals of white matter, cerebrospinal fluid and whole brain. Then bandpass filter was executed with a frequency range of 0.01–0.08 Hz. Finally, the data were smoothed with a Gaussian kernel of 4 × 4 × 4 mm^3^ full width at half maximum (FWHM). These additional rfMRI preprocessing steps were carried out using the SPM12 software (https://www.fil.ion.ucl.ac.uk/spm/software/spm12/).

The downloaded HCP dMRI data had been preprocessed based on the minimal pipelines, including intensity normalization, distortion correction, image motion correction, gradient nonreality correction, and brain extraction (Glasser et al., 2013). Then the spatial normalization parameters from the diffusion space to the MNI space (diffusion‐to‐MNI) were estimated with the following steps: (a) the sMRI data were normalized into the MNI space using Diffeomorphic Anatomical Registration using Exponentiated Lie algebra (DARTEL) algorithms (T1‐to‐MNI); (b) the dMRI were affinely corregistered into the sMRI space (diffusion‐to‐T1); (c) the diffusion‐to‐MNI deformation parameters were calculated by concatenating the two preceding coregistration steps (i.e., diffusion‐to‐T1 and T1‐to‐MNI). These steps were carried out using the FSL 5.0.10 (http://fsl.fmrib.ox.ac.uk/fsl) and SPM12 (https://www.fil.ion.ucl.ac.uk/spm/software/spm12/).

#### 2.3.2 Chinese datasets

The rfMRI data of the Chinese young cohort were preprocessed with the following steps: slice timing to correct for interslice time delay, motion correction to correct for head motion induced displacement, spatial normalization into the MNI space via the sMRI using DARTEL algorithms (Ashburner, 2007), nuisance regression, bandpass filtering, and smoothing. The parameters for the last three steps (including nuisance regression, bandpass filtering, and smoothing) were identical to those for the HCP dataset.

The dMRI data of the two Chinese cohorts were preprocessed with the following steps: eddy current and motion correction (FSL eddy_correct), brain extraction (FSL bet), and diffusion‐to‐MNI spatial normalization. The spatial normalization steps were identical to those for the HCP dataset. For the Chinese elder cohort, the diffusion tensor was additionally estimated using the linear least square model at the native space, and fractional anisotropy (FA) was calculated based on the derived eigenvalues of the diffusion tensor (FSL dtifit). Finally, the FA map was normalized into the MNI space using the diffusion‐to‐MNI parameters. The normalized FA maps of the Chinese elder cohorts were used for test the association between normal aging and the integrity of the right FIC‐dACC structural connectivity. These steps were carried out using the FSL 5.0.10 (http://fsl.fmrib.ox.ac.uk/fsl) and SPM12 (https://www.fil.ion.ucl.ac.uk/spm/software/spm12/).

### 2.4 Definition of SN

**Figure 2:**
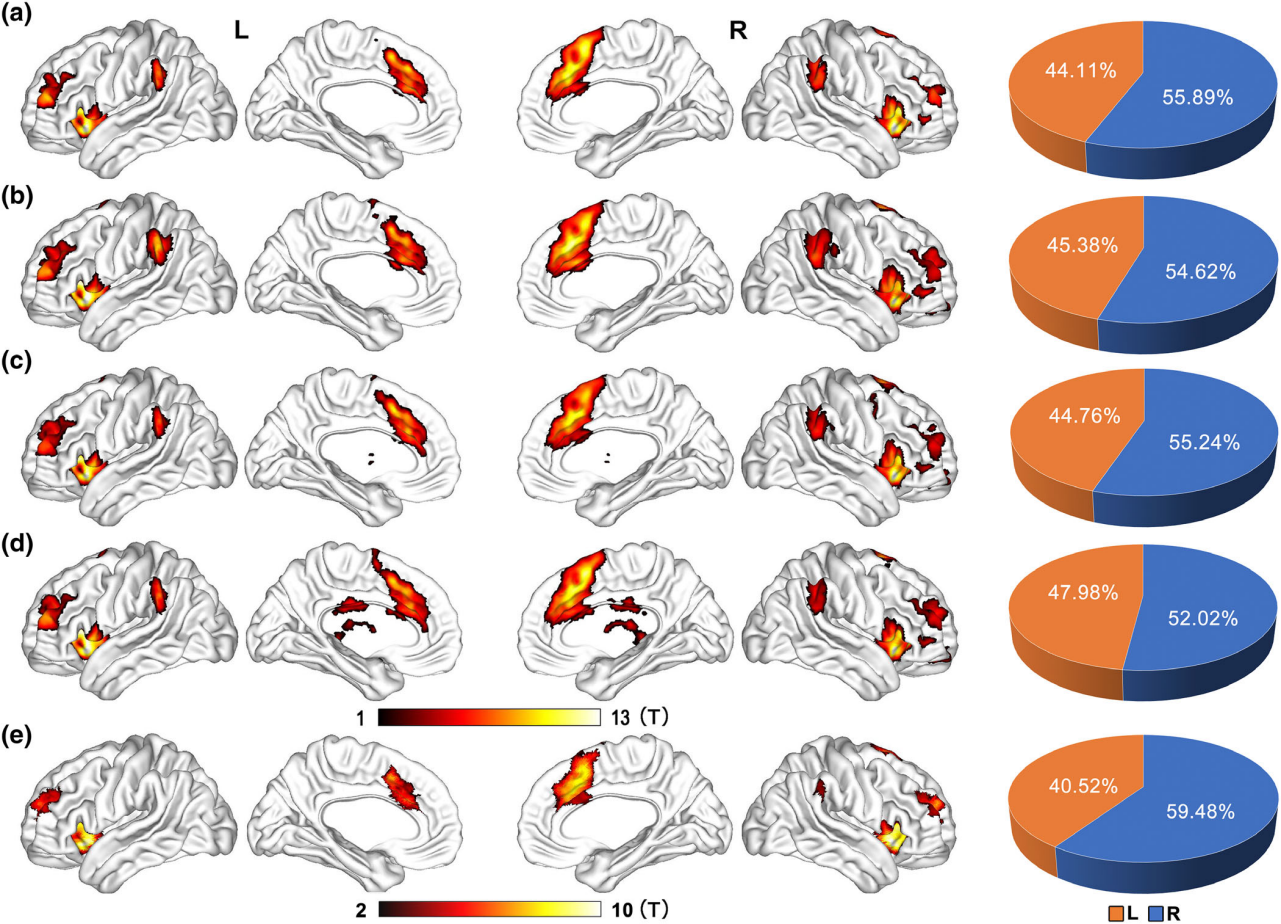
Spatial distribution of the salience network as identified by functional connectivity. Mapping of the SN is reconstructed by conjunction analysis (*q* < 0.001, FDR corrected) of functional connectivity of three SN core hubs (the bilateral FIC and the dACC) from each run of the HCP rfMRI dataset (a–d), and from the Chinese young dataset (e). The color bar represents the *t*‐value of conjunction analysis. The right panels represent the volume ratio of the identified SN in the left (orange) and right (blue) hemisphere, respectively. Abbreviations: dACC, dorsal anterior cingulate cortex; FDR, false discovery rate; FIC, frontoinsular cortex; rfMRI, resting‐state functional magnetic resonance imaging; HCP, human connectome project; SN, salience network

The definition of the SN was carried out firstly on the HCP dataset. The coordinates of three SN hubs were derived from a task‐based fMRI activation map evoked by an auditory event segmentation task from a highly influential article (Sridharan et al., 2008), which reported that this auditory task can evoke strong activation of the core hubs of the SN, including the FIC and dACC; furthermore, the identified SN hubs had been shown to play a critical role in switching between CEN and DMN, a central function of the SN. Three hubs, including the left (MNI coordinates: −32, 24, −6) and right FIC (MNI coordinates: 37, 25, −4), and dACC (MNI coordinates: 4, 30, 30), were extracted from the article. Then, spherical ROIs were defined as voxels contained in 6‐mm radius spheres centered on the three coordinates. Pearson correlation coefficients were calculated between the mean timecourses of each ROI and those of each brain voxel of each run of HCP rfMRI data, which were further converted to *z* values (namely, the FC) using Fisher's *r*‐to‐*z* transformation to improve the normality. Conjunction analyses were carried out on each rfMRI run to identify brain areas that had significantly positive FC with all of the SN ROIs. A false discovery rate (FDR) method with a threshold of *q* < .001 was selected to correct for multiple comparisons (Figure 2). Then, the overlap of the conjunction maps of the four runs was identified as the distribution of functional organization of the SN. The clusters of bilateral FIC and the dACC extracted from the HCP group were used for the following FIC‐dACC structural and FC analyses in both the HCP cohort and the Chinese young cohort. Because the dACC between the left and right hemisphere was merged into one cluster, we manually split it into two independent clusters (left and right dACC) at the midline of the brain.

During validation, the aforementioned SN definition steps were also carried out independently in the Chinese young dataset to repeat the laterality findings of the HCP dataset. Furthermore, because the volume of the extracted core hubs of SN are dominant at the right hemisphere, it is possible that larger ROIs would track a greater number of fibers. To eliminate the possibility that the laterality was caused by bias in seed volumes, we additionally eroded the right FIC and dACC into the same size as the left ones. This step was carried out by automatically up‐thresholding the *t*‐value of the conjunction clusters using a self‐coded MATLAB script, which reduces the cluster size of the right FIC and dACC gradually and final reach the same size as the left ones. This method retains the most significant voxels on the conjunction T map, thus the derived clusters may more specific to represent the cores of the SN.

Finally, to test whether the spatial extent of the SN is lateralized, the volume ratios of the identified SN in the left and right hemisphere in each run of the HCP dataset, and those of the Chinese young dataset were estimated.

### 2.5 Fiber tracking and structural laterality quantification

The structural laterality was evaluated on the HCP and Chinese young dataset, independently. GQI‐based fiber tracking was introduced to construct the structural connectivity between the FIC and dACC clusters in each hemisphere using DSI‐Studio 2017 (http://dsistudio.labsolver.org). GQI is an advanced fiber reconstruction technique developed by Yeh, Wedeen, and Tseng (2010). The GQI‐based software, DSI‐Studio, has been downloaded for 30,000+ times (https://wwww.nitric.org/top/toplist.php?type=downlades). In the ISMRM 2015 Tractography challenge, the GQI‐based fiber tracking method has achieved the highest valid connections (92%) with relatively lower invalid connections among 96 methods from 20 different research groups (Maier‐Hein et al., 2017). Theoretically, the GQI estimates the SDF and can reliably reconstruct multiple fiber orientations within one single voxel (Yeh et al., 2013; Yeh & Tseng, 2010). Unlike the orientation distribution function (ODF) that represents the probability distribution of the diffusion displacement, the SDF quantifies the distribution of spins undergoing diffusion, which is equal to ODF multiplied by the spin density. This method can be generalized into different *q*‐space dataset, such as single‐shell, multi‐shell, and grid sampling schemes. The theoretical basis of the GQI reconstruction is based on the following equation:

(1) $$\mathrm{SDF}\left(r,d\right)=A_{q}L_{\Delta }\sum_{q}W\left(r,q\right)\mathrm{sinc}\left(2\pi \;L_{\Delta }q\cdot d\right)$$

In which *r* represents the voxel coordinate; *d* is the spin diffusion direction, and *q* = *γGδ*/2*π*, in which *γ* is the gyromagnetic ratio, *G* and *δ* are the strength and duration of diffusion encoding gradient; *A* is a constant area term for the quadrature; *L*_Δ_ is the diffusion sampling length; *W*(*r*,*q*) is the diffusion weighted image data.

Then, the SDF of each voxel was fitted directly from the dMRI data using the GQI method (Yeh et al., 2013) for both the HCP and Chinese young cohort, with a diffusion sampling length ratio of 1.25 and diffusion ODF decomposition fraction of 0.05. After SDF estimation, the whole brain fibers were reconstructed using a modified streamline deterministic tracking algorithm and whole brain seeding strategy with the following parameters: step size 0.62 mm, fiber length extent 20–300 mm, threshold for normalized quantitative anisotropy (*n*QA) 0.06, turning angle threshold (45°), and maximum subvoxel search seeds 1,000,000. The whole brain seed was defined as all white matter voxels survived after anisotropy thresholding (*n*QA <0.06). In each of the 1,000,000 tracking iterations, a random subvoxel coordinate within the whole brain seed is selected and fiber tracking algorithm starts from the coordinate and track in two directions until the fiber reaches the brain border. Thus, after finish all the iterations, all possible fibers within the whole brain were generated. Then the fibers that ended in both the dACC and FIC (defined by HCP dataset) were extracted in each subject. The spurious fibers between the dACC and FIC were further manually trimmed based on prior anatomical knowledge by an experienced researcher (W.Q.) (Oishi, Faria, Van Zijl, & Mori, 2010). Then, we calculated the fiber number between the dACC and FIC in each hemisphere. The structural laterality index (SLI) of the SN of each subject was calculated using the following equation:

(2) $$\mathrm{SLI}=\frac{\left(\mathrm{NFR}-\mathrm{NFL}\right)}{\left(\mathrm{NFL}+\mathrm{NFR}\right)}$$

in which the NFL represents the number of fibers between the left FIC and dACC, and the NFR represents number of fibers between the right FIC and dACC.

### 2.6 Functional laterality quantification

The mean timeseries of the FIC and dACC clusters of the SN were extracted from each run of the HCP rfMRI datasets, and from the Chinese dataset. The FC between the two SN hubs in each hemisphere was calculated in an ROI‐wise manner. It should be noted that we calculated the FC based on the unsmoothed rfMRI data, because smoothness may introduce contamination by the contralateral signals in the dACC and bias the laterality calculation. The functional laterality index (FLI) of the SN of each run of each subject was calculated using the following equation:

(3) $$\mathrm{FLI}=\frac{\left(\mathrm{FCR}-\mathrm{FCL}\right)}{\left(\mathrm{FCL}+\mathrm{FCR}\right)}$$

in which the FCL represents the FC between the left FIC and dACC, and the FCR represents the FC between the right FIC and dACC. The FCs and the FLI of the eroded clusters were also calculated.

### 2.7 Population connectivity probability mapping and metric extraction

To characterize the populational trajectory of the FIC‐dACC structural connectivity, the group‐wise trajectory probability map of the FIC‐dACC fibers was generated by averaging the fiber pathway of all of the HCP subjects. Then, a threshold of 50% was introduced on the probability map to generate a group‐wise fiber mask that was used to extract the FA value along the FIC‐dACC fiber trajectory of each Chinese elder subject. The group‐wise fiber probability map of the FIC‐dACC was also generated in the Chinese young cohort to validate the finding in the HCP dataset.

### 2.8 Statistical analysis and validations

To elucidate if the structural and functional laterality exist in the HCP dataset and the Chinese young dataset, One‐sample permutation statistics were carried out on the laterality indices after correcting for multiple comparisons (number of permutations =10,000, FDR correction, *q* < .05). Two‐sample paired permutation statistics were used to compare the differences in fiber number between the right and left hemispheres (number of permutations =10,000, FDR correction, *q* < .05). Two‐sample permutation statistics were used to compared the SLI and FLI between the HCP and Chinese young cohorts (number of permutations =10,000, FDR correction, *q* < .05).

To evaluate the effect of normal aging on the integrity of right FIC‐dACC structural connectivity in the Chinese elder cohort, a partial correlation analysis between the age and FA of the right FIC‐dACC was performed after controlling for the effects of imaging scanners and gender (*p* < .05). All these statistics were carried out using the embedding code in MATLAB R2011a (http://www.mathworks.com).

We introduced the following steps to validate the reliability of the SN laterality (Figure 1). First, two different datasets, the HCP and Chinese young dataset, were used to test whether the functional and structural laterality was repeatable across datasets using the following two different forks: (a) the core hubs of SN (including the FIC and dACC) were defined based on the HCP rfMRI dataset, which were then used to track the fibers in the Chinese young dataset and evaluate the structural laterality (master fork); (b) the SN hubs were independently defined by the rfMRI data of the Chinese young dataset, and they were used to evaluate the functional and structural laterality (supplementary fork). Second, we calculated the functional laterality in each run of the HCP rfMRI datasets, and tested the repeatability of the functional laterality. Finally, because the extracted SN clusters of the right FIC and dACC were larger than those of the left ones, to exclude the possibility that the laterality is caused by bias in seed volumes, the FIC and dACC of the right hemisphere were automatically eroded into the same sizes as the left ones; then the structural laterality was calculated and evaluated repeatedly.

## 3 RESULTS

### 3.1 Functional organization of the SN

Conjunction analysis identified several functional hubs of the SN (*q* < .001, FDR corrected), including the bilateral FIC, dACC, temporal parietal junction (TPJ), and anterior middle frontal gyri (MFG) in the HCP dataset (Figure 2a‐2d, Table S1), and including the bilateral FIC, dACC and anterior MFG, and right TPJ in the Chinese young dataset (Figure 2e, Table S1). The conjunction maps were similar across different runs in the HCP dataset, and were similar between the HCP and Chinese young datasets, indicating a stable functional organization of the SN. Furthermore, pie charts demonstrated a right‐sided laterality in volume ratio of the SN in both the HCP and Chinese young datasets (Figure 2, right panel).

### 3.2 Structural connectivity profile of the SN

**Figure 3:**
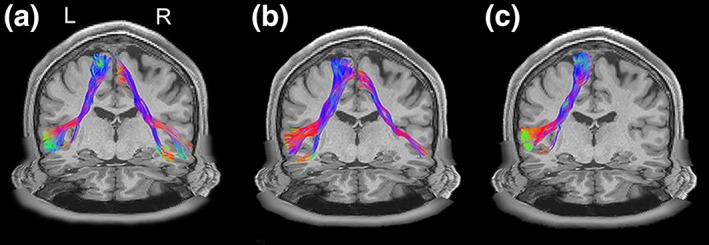
Examples of FIC‐dACC structure connectivity. Three examples of the FIC‐dACC fiber bundles are present: (a) left–right balanced; (b) right‐dominant, and (c) right‐only. All of these examples were obtained from the HCP datasets with generalized q‐sampling imaging reconstruction and deterministic streamline fiber tracking. Abbreviations: dACC, dorsal anterior cingulate cortex; FIC, frontoinsular cortex; HCP, human connectome project

**Figure 4:**
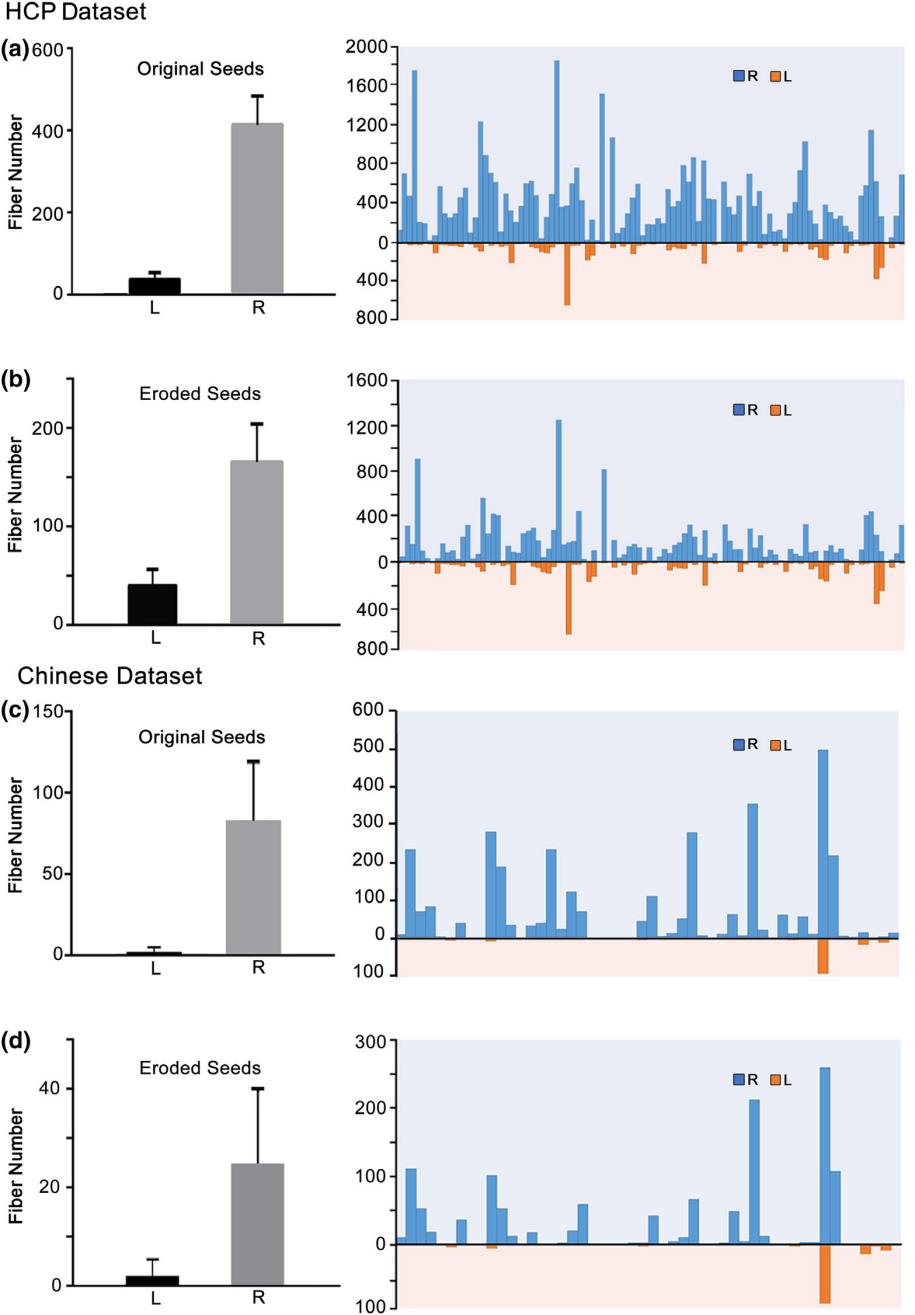
Quantification of the FIC‐dACC structural connectivity in each hemisphere. The fiber numbers of each individual (right panel) are shown and their averages (left panel) are calculated based on: (a) HCP dataset using original FIC and dACC seeds deriving from the conjunction analysis; (b) HCP dataset using the eroded seeds to ensure the cluster sizes between the left and right side are comparable; (c) Chinese dataset using original seeds from the conjunction analysis; and (d) Chinese dataset using the eroded seeds. Abbreviations: dACC, dorsal anterior cingulate cortex; FIC, frontoinsular cortex; HCP, human connectome project

**Figure 5:**
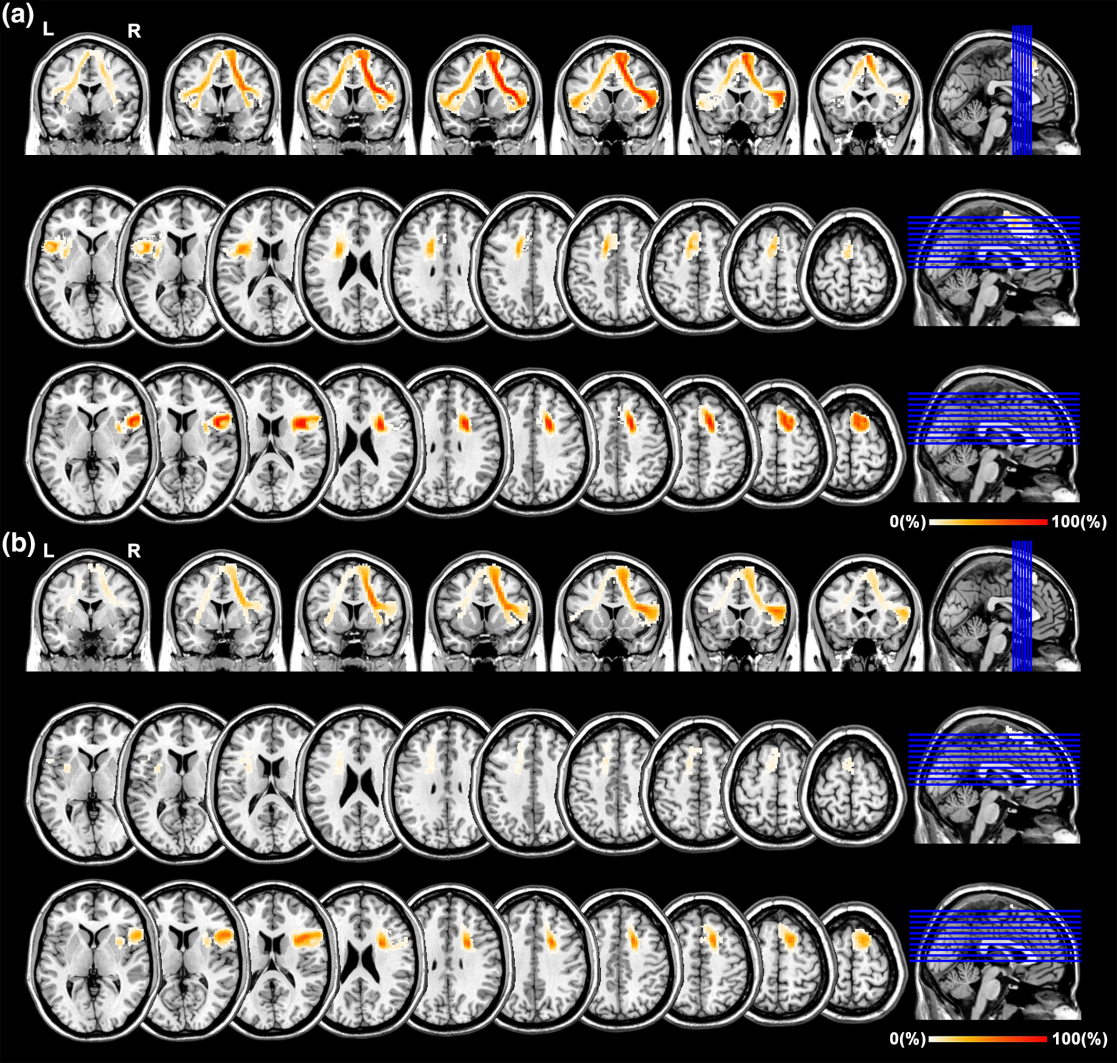
Group probability map of the FIC‐dACC fiber trajectory. The group probability map is created by averaging the individual fiber trajectory of the 100 HCP subjects. The color bar represents the probability value along the FIC‐dACC fiber trajectory. Abbreviations: dACC, dorsal anterior cingulate cortex; FIC, frontoinsular cortex; HCP, human connectome project

An example of the FIC‐dACC structural connectivity can be seen in Figure 3. Deterministic tractography using the hubs defined by the HCP fMRI dataset successfully identified FIC‐dACC fibers bundles in 98/100 (98%) cases in the right hemisphere and 84/100 (84%) cases in the left hemisphere in the HCP dataset, and identified bundles in 38/50 (76%) cases in the right side and 8/50 (16%) cases in the left side in the Chinese young dataset. To validate whether the structural connectivity laterality was influenced by bias in seed volume, the right FIC and dACC seeds was automatically eroded into the same sizes as the left ones. The right FIC‐dACC fibers were also detected in 98/100 (98%) cases for the HCP cohort and in 31/50 (62%) cases for the Chinese young cohort. The average number of FIC‐dACC fibers of the right hemisphere was much higher than that of the left side in both the HCP and Chinese young datasets no matter before or after seed eroding (*p* < .001, permutation test; Figure 4). To characterize the populational trajectory of the FIC‐dACC structural connectivity, the group‐wise trajectory probability map of the FIC‐dACC fibers was generated by averaging the fiber pathway of all of the HCP subjects. As shown in Figure 5, there was also a significant right‐sided dominance in probability distribution of the FIC‐dACC fibers. From cranial to caudal slices, these fibers were projected from the subcortical white matter of the anterior cingulate cortex, then crossed the anterior corona radiate, and finally terminated at the same side FIC region. Notably, the fibers sprouted two branches in the FIC: one branch terminated at the frontal operculum and the other at the anterior insula via the external capsule. The trajectories for the HCP and Chinese young datasets were quite similar, except that the fiber probabilities of the HCP dataset were much higher than those of the Chinese young cohort in both hemispheres (Figure 5).

In the validation procedure, based on the hub definitions using the rfMRI data of the Chinese young cohort, deterministic tractography successfully identified FIC‐dACC fiber bundles in 43/50 (86%) cases in the right side and 8/50 (16%) in the left side in the Chinese young dataset. After seed eroding, the right FIC‐dACC fibers were also detected in 26/50 (52%) cases in the Chinese young cohort (Figure S1). There was also a significant right‐sided dominance in probability distribution of the FIC‐dACC fibers in the Chinese young dataset (Figure S2).

### 3.3 Structural and functional laterality

**Figure 6:**
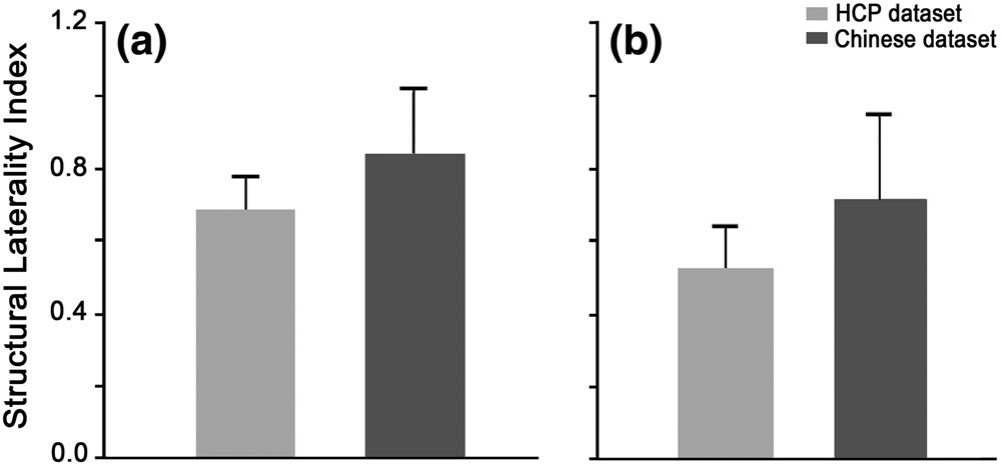
The laterality index of the FIC‐dACC structural connectivity. The structural laterality index of the HCP and Chinese dataset are calculated based on fiber tracking using: (a) the original FIC and dACC seeds deriving from the conjunction analysis and (b) the eroded seeds to ensure the cluster sizes between the left and right side are comparable. Abbreviations: dACC, dorsal anterior cingulate cortex; FIC, frontoinsular cortex; HCP, human connectome project

**Figure 7:**
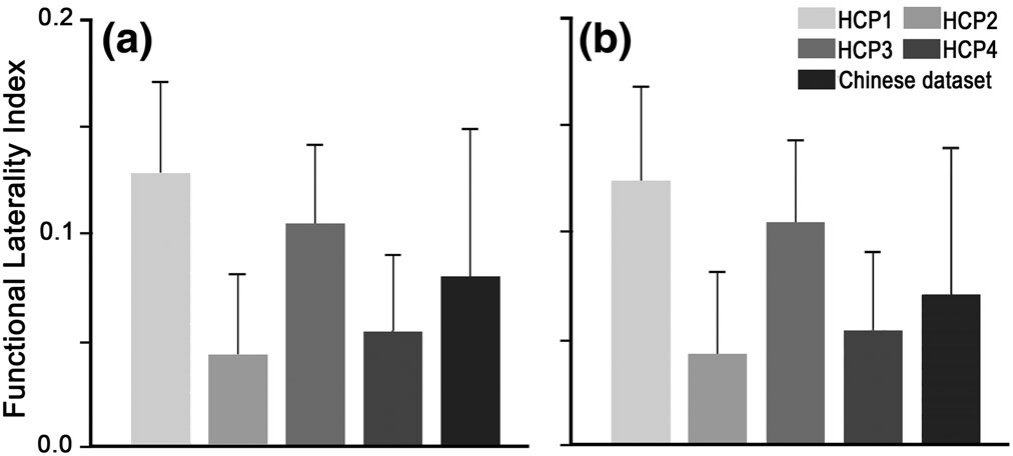
The laterality index of the FIC‐dACC functional connectivity. The functional laterality index of the HCP and Chinese dataset are calculated based on the functional connectivity using: (a) the original FIC and dACC seeds deriving from the conjunction analysis and (b) the eroded seeds to ensure the cluster sizes between the left and right side are comparable. HCP 1–4 represents each of the four runs of the HCP datasets, respectively. Abbreviations: dACC, dorsal anterior cingulate cortex; FIC, frontoinsular cortex; HCP, human connectome project

To quantify the hemisphere dominance of the SN connectivity, we calculated the laterality index of the FIC‐dACC connectivity strength (1 represents fully right laterality, −1 indicates fully left laterality, 0 indicates no laterality). The SLI was 0.727 ± 0.407 and 0.825 ± 0.510 (one‐sample permutation test, *q* < .05, FDR corrected) for the HCP and Chinese dataset, respectively (Figure 6a). The structural laterality survived after correcting for the effect of the seed size (0.528 ± 0.576 for HCP, 0.495 ± 0.638 for Chinese young dataset, *q* < .05, FDR corrected; Figure 6b). The right‐sided laterality of FC also existed in each dataset of the four HCP runs and in the Chinese young dataset (Figure 7a), and survived after correcting for the effect of seed size (*q* < .05, FDR corrected) (Figure 7b). Neither the SLI nor the FLI showed statistical differences between the HCP and Chinese young datasets (two‐sample permutation test, *p* > .05, uncorrected).

In the validation procedure, based on the hub definitions using the rfMRI data of the Chinese young cohort, Chinese young subjects also had significantly positive SLI and FLI (one‐sample permutation test, *q* < .05, FDR corrected). After seed eroding, significant right‐sided laterality was also existing, as revealed by the SLI and FLI (one‐sample permutation test, *q* < .05, FDR corrected; Figure S3).

### 3.4 Effect of aging on the integrity of right FIC‐dACC structural connectivity

**Figure 8:**
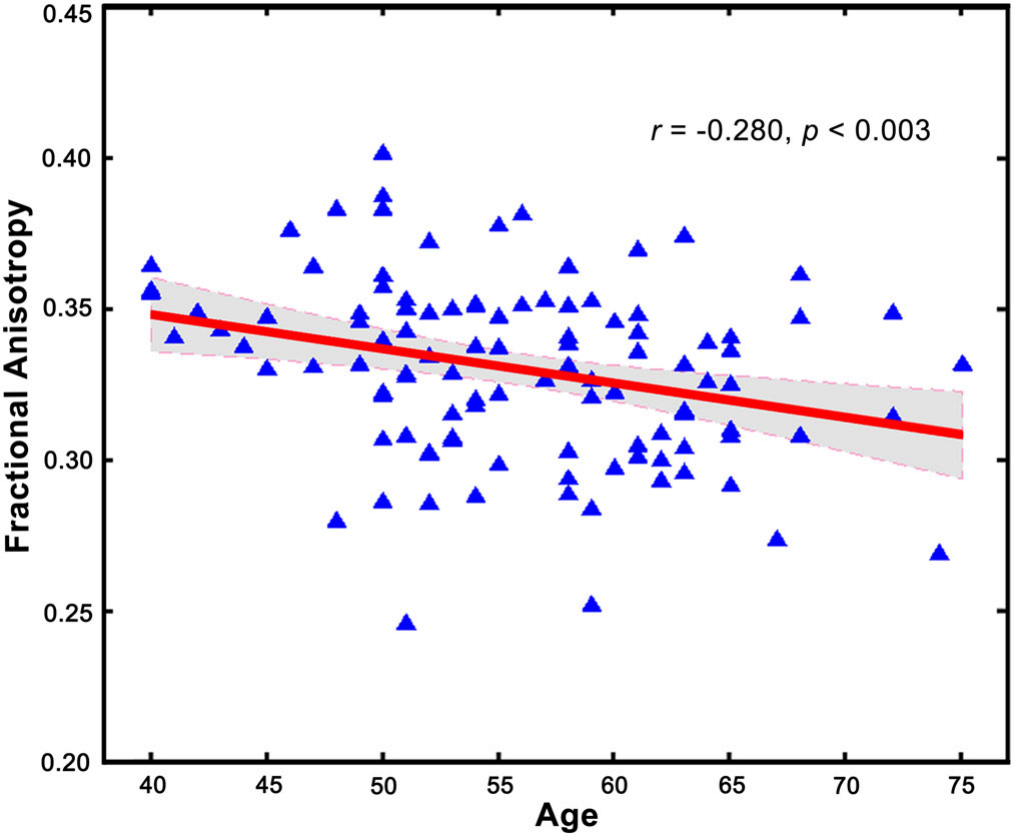
The association between aging and FA of right FIC‐dACC structural connectivity. Partial correlation analysis demonstrated a significant negative association between age and FA of the right FIC‐dACC (*p* < .003). Abbreviations: dACC, dorsal anterior cingulate cortex; FA, fractional anisotropy; FIC, frontoinsular cortex [Color figure can be viewed at http://wileyonlinelibrary.com]

After controlling for the effect of imaging scanners and gender, partial correlation analysis demonstrated significant negative association between age and FA of the right FIC‐dACC (*r* = −0.280, *p* < .003), suggesting the presence of decreased integrity in structural connectivity of SN for the elder (Figure 8).

## 4 DISCUSSION

In the present study, to elucidate the potential connectivity lateralization of the salience network, the structural and FC between the FIC and dACC, two core hubs of salience network, were constructed using multimodal magnetic resonance data from independent datasets. We found a significantly right‐sided laterality in both functional and structural connectivity between the FIC and dACC. Furthermore, the laterality mode is replicable in different datasets, and it cannot be explained by differences in seed size. Finally, we found a significant age effect on the integrity of right FIC‐dACC structural connectivity. The right‐sided laterality of structural organization of the salience network may help us to better understand the functional roles of salience network in the human brain.

Converging results from previous studies suggested that the functional organization of the FIC and dACC is right lateralized. For example, the right rather than the left FIC was predominantly activated when participants perceive salient auditory event boundaries (Sridharan et al., 2008) or perceive their own heartbeat (Ronchi et al., 2015; Zaki, Davis, & Ochsner, 2012). The right dACC was also predominantly activated in monitoring cognitive conflict (Lutcke & Frahm, 2008) and initializing prediction on novel stimuli (Weiss et al., 2018). Besides local activation, Granger causal analyses revealed that the right FIC predominantly exerted strong causal net outflow to the CEN and DMN under either task‐ or resting‐state conditions (Sridharan et al., 2008). Dynamic causal modeling analyses also demonstrated that salient input into the salience network was most likely via the right anterior insular rather than the left one during Simon task (Ham et al., 2013). Furthermore, FC (Cauda et al., 2011) and ICA (Seeley et al., 2007) identified stronger and broader intrinsic functional couplings in the right FIC and dACC of the salience network than those in the left side. In consistence with the above‐mentioned studies, we demonstrated that the functional laterality of the salience network is much stable at resting‐state, indicating FC laterality between the FIC and dACC is a reliable and important representation of salience network.

One of the most important findings of the present study was the right‐laterality of the structural connectivity between the FIC and dACC. Early evidence of the structural relationships between the FIC and dACC was derived from the finding of VENs (Economo, 1926). Several preceding studies identified that the VENs, a type of large bipolar projection neurons in layer V, specifically locate in the dACC and FIC (Allman et al., 2010; Cobos & Seeley, 2015). Through the VEN projections, these two regions have interconnections with each other, and connect with multiple brain regions, such as the prefrontal cortex, insular and amygdala, and so on (Allman et al., 2010; Craig, 2009; Kim et al., 2016; Seeley et al., 2012). Moreover, it is interesting to note that the amount of VENs are much higher in the right FIC and dACC than in the left ones in both the postnatal apes and humans (Allman et al., 2010; Evrard et al., 2012). Thus, we speculated that the right laterality of the FIC‐dACC connectivity found in the present study may be a macroscopic representation of the VEN projections.

Early studies have demonstrated that the FC between brain areas is closely associated with direct or indirect structural connectivity (Honey et al., 2009; Teipel et al., 2010). Thus, the structural laterality of FIC‐dACC connectivity found in the present study may be an important representation of the functional laterality of the salience network, which was supported by a recent study showing that the structural connectivity can characterize the functional laterality of language network (Sreedharan et al., 2015). It should be noted that the laterality index of FIC‐dACC structural connectivity in the present study were much higher than those of the FC (see Figures 6 and 7), implying that structural connectivity is more sensitive in representing the laterality of the salience network. The lower laterality of FC may be explained by the fact that FC can be influenced by more complex factors than the structural connectivity, such as the indirect synaptic connections, neuronal dynamics, and neuromodulation (Bargmann & Marder, 2013).

As the main components of the salience network, the dACC and the FIC, undertake to recognize the most salient events among a large number of internal and external stimuli to guide cognitive processing (Gradin et al., 2013; Luo et al., 2014; Manoliu et al., 2013). For example, the right FIC was reported to be dominantly involved in awareness, errors, and emotional feedback (Craig, 2009; Ronchi et al., 2015; Ullsperger, Harsay, Wessel, & Ridderinkhof, 2010). The right FIC was also preferentially associated with aversive, egocentric, and negative affects relating to sympathetic activity (Allman et al., 2010; Craig, 2009). In addition, the right FIC is considered as a core hub of right‐lateralized ventral attention network, which is involved in stimulus‐driven visual‐spatial processing (Corbetta, Patel, & Shulman, 2008; Corbetta & Shulman, 2011). Lesion‐symptom mapping analyses demonstrated that damage to the right FIC can cause severe unilateral visual‐spatial neglect (Verdon, Schwartz, Lovblad, Hauert, & Vuilleumier, 2010). Because a greater number of fibers between the FIC and dACC helps to information communications between them, we speculated that the right‐laterality of the FIC‐dACC structural connectivity is crucial for integrating and transferring salient information. Our hypothesis was supported by an early dynamic causal modeling analysis demonstrating that the right FIC may be the first place to receive salient inputs and transfer filtered information to other nodes of the salience network (Ham et al., 2013). Another supporting study also demonstrated that the right FIC plays a critical and causal role in switching between the CEN and the DMN (Menon & Uddin, 2010; Sridharan et al., 2008): during salient‐evoked tasks, the casual flows from the right FIC to the dACC and CEN are strengthened, while those to the DMN are suppressed; under a task‐free resting‐state, the flows from the right FIC to the CEN are suppressed, while those to the DMN are strengthened. Our hypothesis can also be explained by the electrophysiological characteristics of VENs: They have larger size in soma and projections than the neighboring pyramid neurons (Evrard et al., 2012), thus they own higher conductivity, which facilitate electrophysiological signal transmission between neurons. Accordantly, Allman, Watson, Tetreault, and Hakeem (2005) proposed that the VENs and related circuitry are involved in immediate effortless awareness rather than the engagement of deliberative processes. The right predominance of VENs amount in the FIC/dACC can explain the laterality in cognitive role of the salience network: it provides broader and faster pathway to immediately process the salient stimuli within the salience network and to transmit them to other brain networks. Accordingly, it is not difficult to infer why do the right FIC and dACC were frequently reported in participating in multiple types of rapid cognitive activities at the early stage, such as awareness, stimulus‐driven attention, and sympathetic activities, and so on, in comparison to the left side ones that were related with the slow neural responses such as parasympathetic control (Evrard et al., 2012; Guo et al., 2016). Thus, the structural connectivity between the right FIC and the dACC may be the candidate neural pathway to transfer salient information to other brain networks.

In the present study, we found a significantly negative correlation between age and integrity of the right FIC‐dACC structural connectivity. Early studies have indicated that the cognitive functions relating to salient processing are reduced in the aging, such as alerting (Jennings et al., 2007), task‐switching (Verhaeghen & Cerella, 2002), and conflict processing (West, 2004), and so on. In accordance with the cognitive reduction, several studies reported that FC within the salience network, and between the right FIC and the CEN/DMN was reduced with normal aging (He et al., 2013; He et al., 2014). Besides, the GMV of the right FIC and dACC was also reduced in the elder (He et al., 2014). Our finding provided additional evidence that normal aging can also influence the structural connectivity of the salience network. Several recent studies have reported that the atrophy of the dACC/FIC was associated with loss of VENs in the frontotemporal dementia and Alzheimer's disease; furthermore, the loss of VENs was significantly correlated with cognitive deficits (Gefen et al., 2018; Kim et al., 2016). Thus, we speculated that normal aging may also induce the loss of VENs and their projections. However, the causal relationships between normal aging, integrity of VENs, and cognitive functions are still unknown, which should be clarified in the future.

In summary, based on multiple validation procedures, the present study found a remarkable and stable right‐sided laterality of structural connectivity between the FIC and dACC, which may help us to better understand the functional roles of the salience network in the normal human brain. Furthermore, the right FIC‐dACC structural connectivity may be a potential biomarker to monitor the degeneration of the salience network during normal aging.

## CONFLICT OF INTERESTS

The authors declare no competing financial interests.

## Supporting information


**Table S1** Summary of the cluster peaks during conjunction analyses
**Figure S1. Quantification of the FIC‐dACC structural connectivity in each hemisphere based on the Chinese young dataset**
The FIC and dACC was defined using conjunction analysis based on the rfMRI data of the Chinese young cohort. The fiber numbers of each individual (right panel) are shown and their average values (left panel) are calculated based on: a) the Chinese young dataset using original seeds from the conjunction analysis; b) the Chinese dataset using the eroded seeds. Abbreviations: dACC = dorsal anterior cingulate cortex; FIC = frontoinsular cortex.
**Figure S2. Group probability map of the FIC‐dACC fiber trajectory based on the Chinese Young dataset**
The fiber trajectory of each subject was tracked using the seeds defined by the Chines young rfMRI dataset. The group probability map is created by averaging the individual fiber trajectory of the 50 Chinese young subjects. The color bar represents the probability value of FIC‐dACC fiber trajectory. Abbreviations: dACC = dorsal anterior cingulate cortex; FIC = frontoinsular cortex.
**Figure S3. The laterality index of the FIC‐dACC structural and functional connectivity based on the Chinese Young dataset**
The structural (a) and functional (b) laterality index of the Chinese young dataset are calculated based on fiber tracking and functional connectivity, respectively. The black bar represents the original FIC and dACC seeds deriving from the conjunction analysis, and the gray bar represents the eroded seeds after correcting the effect of seed size. Abbreviations: dACC = dorsal anterior cingulate cortex; FIC = frontoinsular cortex.

## Data Availability

The HCP dataset that support the findings of this study are available by Human Connectome Project at https://www.humanconnectome.org/study/hcp-young-adult. In addition, the Chinese datasets that support the findings of this study are available on request from the corresponding author.
