# Supplementary material for: Structural connectivity profile supports laterality of the salience network
Source: Hum Brain Mapp. 2019 Aug 21;40(18):5242–55. doi: 10.1002/hbm.24769 (PMC6864895; doi:10.1002/hbm.24769)
Supplement: Supplementary file 1 — Table S1 Summary of the cluster peaks during conjunction analyses Figure S1. Quantification of the FIC‐dACC structural connectivity in each hemisphere based on the Chinese young dataset The FIC and dACC was defined using conjunction analysis based on the rfMRI data of the Chinese young cohort. The fiber numbers of each individual (right panel) are shown and their average values (left panel) are calculated based on: a) the Chinese young dataset using original seeds from the conjunction analysis; b) the Chinese dataset using the eroded seeds. Abbreviations: dACC = dorsal anterior cingulate cortex; FIC = frontoinsular cortex. Figure S2. Group probability map of the FIC‐dACC fiber trajectory based on the Chinese Young dataset The fiber trajectory of each subject was tracked using the seeds defined by the Chines young rfMRI dataset. The group probability map is created by averaging the individual fiber trajectory of the 50 Chinese young subjects. The color bar represents the probability value of FIC‐dACC fiber trajectory. Abbreviations: dACC = dorsal anterior cingulate cortex; FIC = frontoinsular cortex. Figure S3. The laterality index of the FIC‐dACC structural and functional connectivity based on the Chinese Young dataset The structural (a) and functional (b) laterality index of the Chinese young dataset are calculated based on fiber tracking and functional connectivity, respectively. The black bar represents the original FIC and dACC seeds deriving from the conjunction analysis, and the gray bar represents the eroded seeds after correcting the effect of seed size. Abbreviations: dACC = dorsal anterior cingulate cortex; FIC = frontoinsular cortex. [file HBM-40-5242-s001.docx]

***Supplementary Information***

**Full title: Structural connectivity profile supports laterality of the salience network**

Yaodan Zhang^1^, Xinjun Suo^1^, Hao Ding^1,2^, Meng Liang^1,2^, Chunshui Yu^1^, Wen Qin^1^

1 From the Departments of Radiology and Tianjin Key Laboratory of Functional Imaging, Tianjin Medical University General Hospital, Tianjin 300052, China

2 School of Medical Imaging, Tianjin Medical University, Tianjin 300070, China

**Correspondence to:** Wen Qin, MD, Department of Radiology, Tianjin Medical University General Hospital, No. 154, Anshan Road, Heping District, Tianjin 300052, China; E-mail: wayne.wenqin@gmail.com. Phone: +86-22-60363760; Fax: +86-22-60362206

**Supplementary Tables**

**Table S1: Summary of the cluster peaks during conjunction analyses**

| Cohorts | Runs | Regions | R/L | Peak MNI coordinates, (mm) | t-values |
| --- | --- | --- | --- | --- | --- |
| HCP | Run1 | FIC | R | 42, 18, -6 | 18.56 |
|  |  |  | L | -44, 16, -4 | 18.22 |
|  |  | dACC | R/L | 0, 16, 52 | 14.16 |
|  | Run2 | FIC | R | 36, 18, -10 | 17.22 |
|  |  |  | L | -42, 16, -6 | 18.64 |
|  |  | dACC | R/L | 0, 22, 44 | 14.01 |
|  | Run3 | FIC | R | 34, 18, -8 | 17.66 |
|  |  |  | L | -46, 16, -6 | 18.61 |
|  |  | dACC | R/L | 0, 16, 52 | 12.88 |
|  | Run4 | FIC | R | 44, 18, -6 | 17.68 |
|  |  |  | L | -42, 16, -4 | 20.87 |
|  |  | dACC | R/L | 0, 16, 50 | 14.38 |
| Chinese young | Run1 | FIC | R | 33, 18, -6 | 11.29 |
|  |  |  | L | -36, 15, -6 | 11.16 |
|  |  | dACC | R/L | 3, 15, 51 | 8.76 |

Notes: dACC = dorsal anterior cingulate cortex FIC = frontoinsular cortex;

**Supplementary Figures**

**
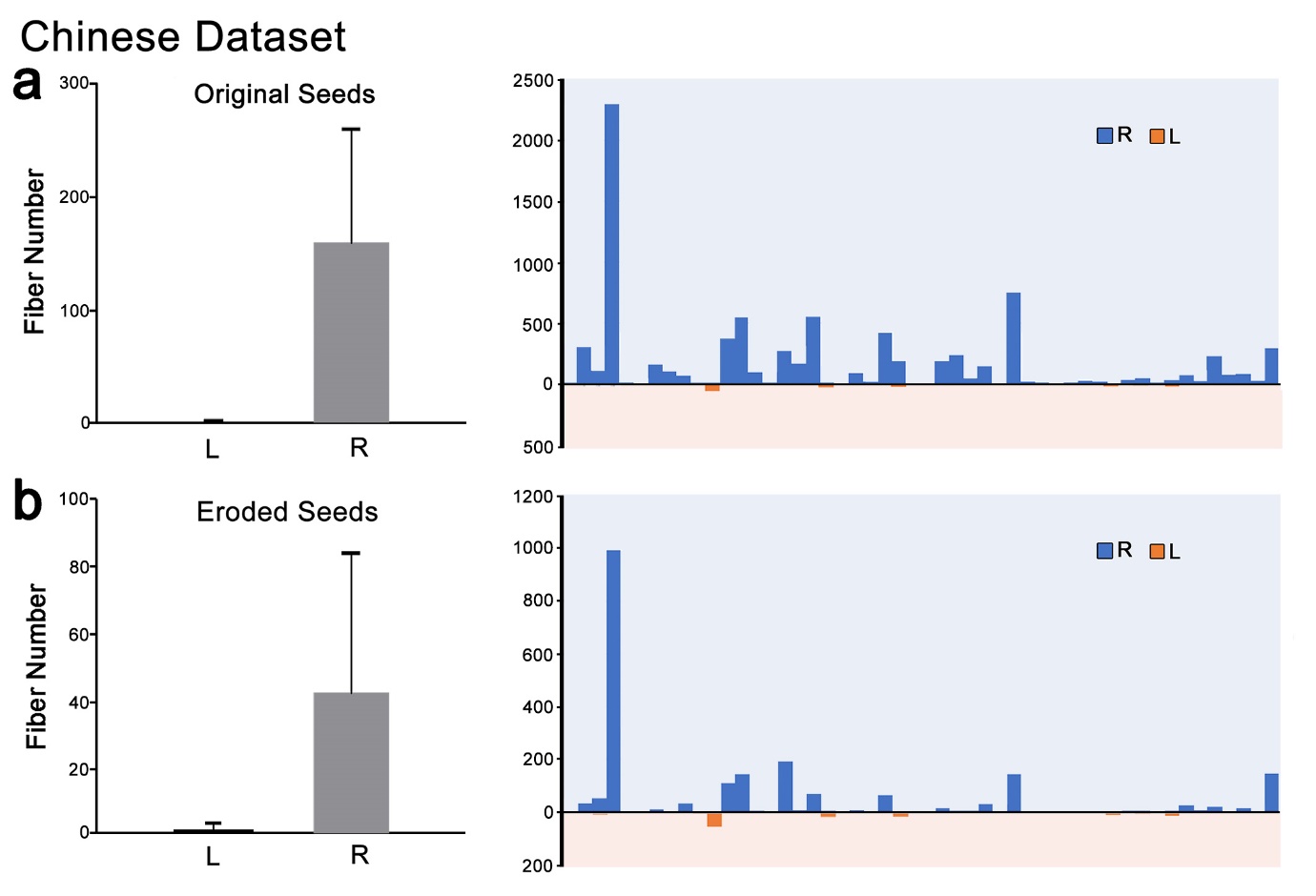
**

**Figure S1. Quantification of the FIC-dACC structural connectivity in each hemisphere based on the Chinese young dataset**

The FIC and dACC was defined using conjunction analysis based on the rfMRI data of the Chinese young cohort. The fiber numbers of each individual (right panel) are shown and their average values (left panel) are calculated based on: a) the Chinese young dataset using original seeds from the conjunction analysis; b) the Chinese dataset using the eroded seeds. Abbreviations: dACC = dorsal anterior cingulate cortex; FIC = frontoinsular cortex.

**
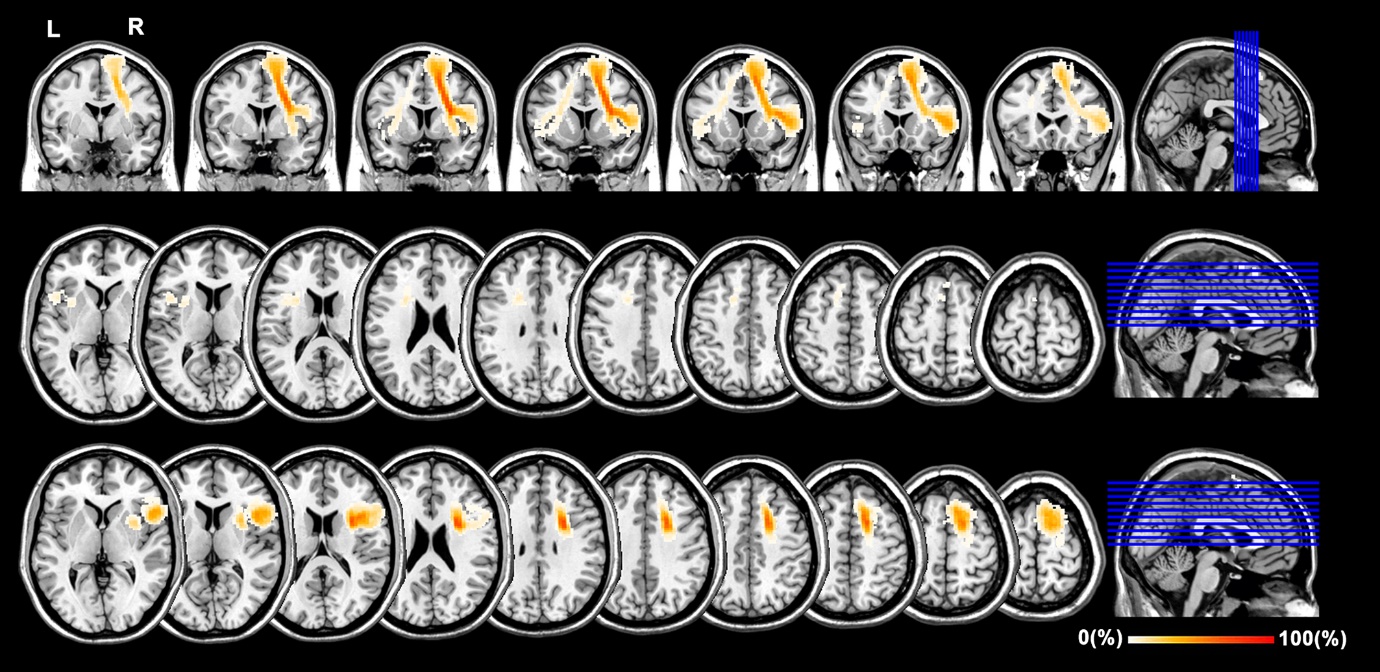
**

**Figure S2. Group probability map of the FIC-dACC fiber trajectory**

The fiber trajectory of each subject was tracked using the seeds defined by the Chines young rfMRI dataset. The group probability map is created by averaging the individual fiber trajectory of the 50 Chinese young subjects. The color bar represents the probability value of FIC-dACC fiber trajectory. Abbreviations: dACC = dorsal anterior cingulate cortex; FIC = frontoinsular cortex.

**
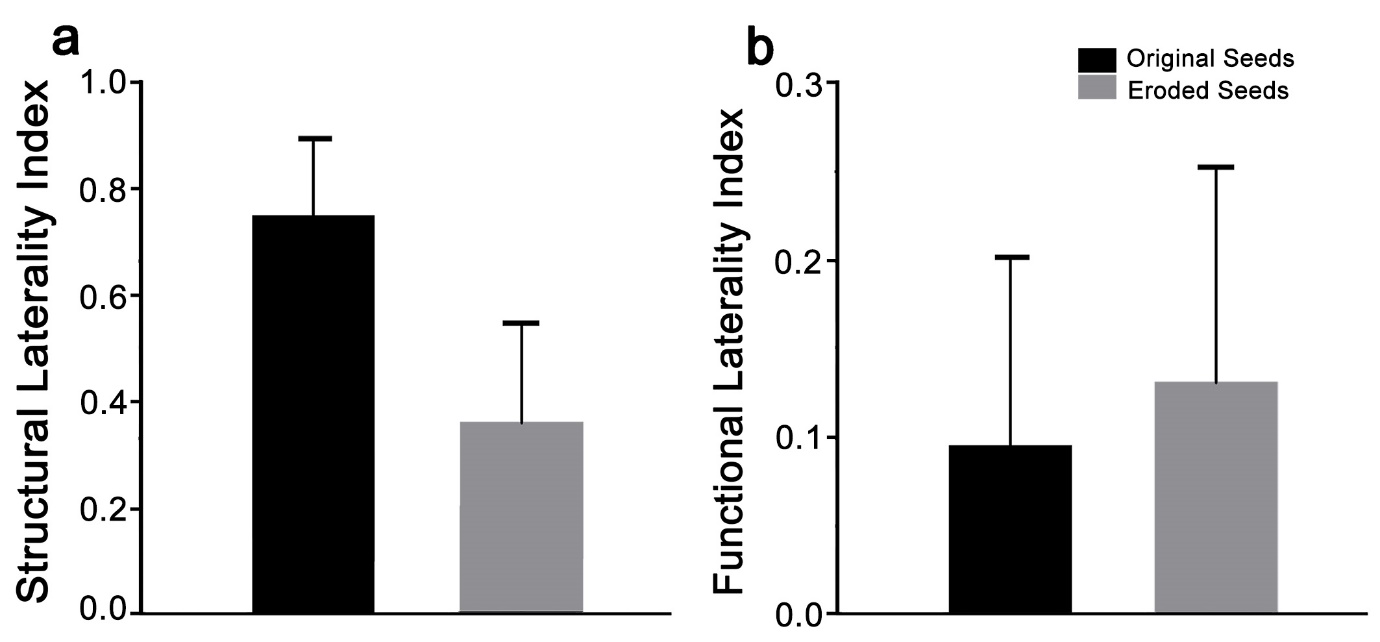
**

**Figure S3. The laterality index of the FIC-dACC structural and functional connectivity**

The structural (a) and functional (b) laterality index of the Chinese young dataset are calculated based on fiber tracking and functional connectivity, respectively. The black bar represents the original FIC and dACC seeds deriving from the conjunction analysis, and the grey bar represents the eroded seeds after correcting the effect of seed size. Abbreviations: dACC = dorsal anterior cingulate cortex; FIC = frontoinsular cortex.
